# Induction Chemotherapy Followed by Radiotherapy versus Concurrent Chemoradiotherapy in elderly patients with nasopharyngeal carcinoma: finding from a propensity-matched analysis

**DOI:** 10.1186/s12885-016-2661-y

**Published:** 2016-08-30

**Authors:** Qi Zeng, Jie Wang, Xing Lv, Jie Li, Li-Jie Yin, Yan-Qun Xiang, Xiang Guo

**Affiliations:** 1 State Key Laboratory of Oncology in South China, Collaborative Innovation Center for Cancer Medicine, Guangzhou, 510060 China; 2 Department of Interventional Oncology, Sun Yat-sen University Cancer Center, Guangzhou, 510060 China; 3 Department of Radiation Oncology, Dalian Municipal Central Hospital, Dalian, 116033 China; 4 Department of Nasopharyngeal Carcinoma, Sun Yat-sen University Cancer Center, 651 Dongfeng Road East, Guangzhou, 510060 People’s Republic of China; 5 Department of Breast and Thyroid Surgery, The First Affiliated Hospital of Sun Yat-Sen University, Guangzhou, Guangdong 510080 People’s Republic of China

**Keywords:** Nasopharyngeal carcinoma, Elderly, Chemo-radiotherapy, Survival

## Abstract

**Background:**

To date, no guideline is proposed for elderly nasopharyngeal carcinoma (NPC) due to lack of prospective clinical trials. The present study comparing the survivals and toxicities in elderly NPC patients received either induction chemotherapy followed by radiotherapy(IC + RT) or concurrent chemoradiotherapy (CCRT) was therefore undertaken to provide a more accurate basis for future clinical practice.

**Methods:**

The eligible elderly NPC patients were retrospectively enrolled. Propensity score matching generated a matched cohort (1:2) composed from CCRT and IC + RT groups. The survivals and treatment-induced toxicities were compared between two groups. Multivariable analysis was carried to identify significant prognostic factors.

**Results:**

The 5-year overall survival, cancer-specific survival, locoregional failure-free survival, distant failure-free survival for all patients were 58.3 %, 62.7 %, 88.7 %, 83.0 %, respectively. No significant survival differences were found between CCRT and IC + RT groups in the propensity-matched cohort. In comparison with the patients who received IC + RT, patients who underwent CCRT were associated with more severe acute toxicities including leucopenia (30 % vs. 6.8 %, *P* = 0.005), anemia (20 % vs. 4.1 %, *P* = 0.027), mucositis (63.3 % vs. 34.2 %, *P* = 0.007), weight loss (23.4 % vs. 4.1 %, *P* = 0.009). Basicranial bone involvement was an independent prognostic factor that predicted all-cause death (HR = 0.553, 95 % CI 0.329–0.929; *P* = 0.025) and cancer specific death (HR = 0.558, 95 % CI 0.321–0.969; *P* = 0.038) in elderly patients.

**Conclusions:**

In the context of no guideline for elderly NPC, the present study suggested IC + RT should be a preferable modality compared with CCRT, with similar treatment outcomes but less acute toxicities.

**Electronic supplementary material:**

The online version of this article (doi:10.1186/s12885-016-2661-y) contains supplementary material, which is available to authorized users.

## Background

Nasopharyngeal carcinoma (NPC) is a special head and neck cancer in terms of its epidemiology, etiology, clinical presentation, and prognostic factors [1]. The incidence of NPC is increasing with age in the endemic areas, with a peak and subsequently an earlier decline in age-incidence (in middle-age, ages 45–60 years) than seen in any low-risk population [2]. Elderly NPC patients (age ≥ 60 years) constitutes about 13.8 % (1310/9527) of all NPC [3, 4]. To date, the treatment for geriatric NPC patients generally follows guidelines tailored for non-elderly patients, but the elderly are usually excluded from prospective clinical trials because of restrictive selection criteria. The development of prospective trials for elderly patients has been hindered by the rarity of patients and accrual difficulties due to the prevalence of comorbidities and decreasing organ function in elderly patients. When a prospective design is difficult to achieve, the rigorously designed retrospective study is of paramount importance in the light of evidence that NPC has certain distinctive characteristics when it occurs in elderly patients [4].

A retrospectively matched cohort study [3] of chemoradiotherapy versus radiotherapy alone in elderly NPC patients from our institute was published in January 2015. In this study, patients received combined chemoradiotherapy, which defined as induction chemotherapy followed by radiotherapy(IC + RT) or concurrent chemoradiotherapy(CCRT), have presented significantly better survival compared with patients received RT alone. Moreover, a 2013 matched analysis also showed CCRT significantly improved the survival in elderly NPC [5]. Thus, we were interested to determine which treatment modality (IC + RT or CCRT) was the optimal treatment strategy for elderly NPC patients. According to previous studies in non-elderly patients [6, 7], we hypothesized that no significant difference of survival will be observed between IC + RT and CCRT groups in elderly patients, but more severe treatment-induced toxicities in CCRT group. If our hypothesis is correct, we propose sequential chemoradiotherapy (IC + RT) should be recommended for elderly NPC patients in view of poorer tolerance to CCRT in elderly patients as opposed to younger ones.

This present study was therefore undertaken to compare the survivals and treatment-induced toxicities between IC + RT and CCRT groups using a propensity-matched analysis in elderly NPC patients (age ≥ 60 years).

## Methods

From January 1998 and December 2003, the patients selected consecutively in our institute met the following criteria: (i) biopsy-proven, previously untreated WHO II or III NPC ; (ii) elderly patient who is 60 years or older; (iii) no second primary tumors; (iv) patients without systemic metastasis; (v) patients received definitive radiotherapy. The study was approved by the Clinical Research Ethics Committee of Sun Yat-sen University Cancer Center. It was a retrospective analysis of routine data and thus we were granted a waiver of individual informed consent. All patients were evaluated by the following examinations before treatment: complete patient history, physical examination, CT or MRI of the neck and nasopharynx, chest radiography, abdominal ultrasonography, and acquisition of whole body bone scans by single photon emission computed tomography (ECT). All patients were restaged according to the sixth edition AJCC/UICC staging system.

### Radiotherapy

All patients received external beam RT by conventional fractionation; Details of RT technique in our cancer center have been reported previously [3]. To put it simply, 64–72 Gy (in 6.5–7 weeks) were delivered to the primary tumor, 60–66 Gy to clinically involved nodes, and 48–50 Gy to uninvolved cervical and supraclavicular areas. Patients with involvement of the skull base were delivered a booster dose (8 to 10 Gy per four to five fractions).

### Chemotherapy

The induction or adjuvant chemotherapy (AC) regimen was mainly a combination of cisplatin and 5-fluorouracil (5-Fu), with cisplatin (30 mg intravenously) given on Day 1–5 and 5-fluorouracil (750 mg intravenously) on Days 1–5, repeated every 3 weeks. The concurrent chemotherapy regimen was mainly cisplatin alone, with cisplatin (30–40 mg/m2 on Day 1) given intravenously weekly or cisplatin (80–100 mg/m2) given intravenously 3-weekly. Dose modification was applied, if needed, at doctor’s discretion.

### Patient assessment and follow-up

After treatment, patients were assessed every 3 months by the first 3 years, and every 6 months thereafter until the fifth year. The local recurrences were diagnosed on MRI or CT scanning or by fiber optic endoscopy and biopsy. Regional recurrences were diagnosed by physical examination or MRI/intensive CT scans; irresolute cases were confirmed by fine-needle aspiration. Distant metastases were diagnosed by combined modalities including CT or MR, bone scan, abdominal ultrasonography, and chest x-ray. Chemotherapy-related toxicities were graded according to the Common Terminology Criteria for Adverse Events (CTCAE) v4.0 [8]. Acute and late RT-related toxicities were graded using the Radiation Morbidity Scoring Criteria of the Radiation Therapy Oncology Group [9]. Late toxicities referred to symptoms that occurred or continued beyond 90 days since the commencement of RT.

### Statistical analysis

The primary end points were overall survival (OS), cancer-specific survival (CSS). The secondary end points were local-regional failure-free survival (LR-FFS), and distant failure-free survival (D-FFS). All intervals were calculated from the date of beginning therapy. OS was defined as the time until death from any causes. CSS referred to the time until death from NPC. LR-FFS was defined as the time until the first recurrence in the cervical and/or nasopharyngeal region after radiotherapy. D-FFS was defined as the time until distant metastasis.

Baseline characteristics of patients in the two groups were accessed using descriptive statistics. The statistical results were presented as the mean ± standard deviation or percentages. Given the differences in the baseline characteristics between the two groups, propensity-score matching was used to identify the cohort of patients with similar baseline characteristics. Matching was performed with the use of a 1:2 matching protocol (nearest-neighbor) for CCRT and IC + RT groups. The matching covariates consisted of age, gender, T classification, N classification, RT dose to nasopharynx and involved cervical lymph node, RT time, cranial nerve involvement, basicranial bone involvement, and family history. Survival analysis was carried out using the Kaplan–Meier method and compared with the log-rank test. The median follow-up time was calculated using the reverse KM estimator [10]. Univariate analyses with the unadjusted Cox proportional hazards model were performed to calculate the hazard ratio (HR). Multivariate analyses using the Cox proportional hazards model were performed to identify independent prognostic factors through the backward elimination. A two-sided P-value of less than 0.05 was taken as statistically significant. The statistical analyses were performed using SPSS version 19.0 (SPSS, Inc., an IBM Company; Chicago, IL, USA). In addition, the propensity-matched analysis was performed using the MatchIt package [11] in R Statistical Software (version 3.1.3; R Foundation for Statistical Computing, Vienna, Austria).

## Results

### Baseline characteristics, survival and patterns of treatment failure in the entire patient

Between January 1998 and December 2003, a total of 498 eligible elderly patients were included in this present study, with a median age of 65 years (60–84 years). The ratio of male to female was 4.53:1, with 408 males and 90 females. The clinical stage distribution was: stage I, 23 (4.6 %); stage II, 127 (25.5 %); stage III, 185 (37.1 %), and stage IVa 163 (32.7 %). In total, 171 (34.3 %) patients were treated with combined chemo-radiotherapy (CRT) and 327 (65.7 %) received radiotherapy (RT) alone. The reverse KM estimate of the median follow-up was 64.7 months (95 % CI: 62.87–66.52 months). The median OS time was 74.6 months. 46 (9.2 %) patients developed locoregional relapse, 78 (15.7 %) developed distant metastases, and 212 (42.6 %) died. The 1-, 3- and 5-year survival rates for the entire group were as follows: OS, 99.8 %, 70.2 % and 58.3 %; CSS, 99.8 % , 72.5 % and 62.7 %; LR-FFS, 99.6 %, 91.5 % and 88.7 %; and D-FFS, 99.8 % , 85.4 % and 83.0 %.

### Treatment Exposure

One hundred seventy-one patients received combined chemo-radiotherapy. In which, 111 cases received IC, only 73 cases completed a full course of two cycles of IC; 44 cases received CCRT, only 30 cases completed 3-weekly concurrent regimens for three cycles or weekly CCRT for at least five cycles; 15 cases received IC + CCRT/AC, only 13 cases completed at least three cycles. Additionally, just 1 case received one cycle of AC. An analysis of IC delivery found patients received fixed lower total doses of each chemotherapeutic drug irrespective of body surface area, primarily as a result of arbitrary dose modification of chemotherapy owing to fear of excessive side-effects. With respect of CCRT, 22.7 % (10/44) patients received decreased doses of cisplatin. The mean total dose of cisplatin was 249 mg vs. 200 mg (*p* = 0.046) between patients received IC + RT and patients received CCRT. These results showed patients received higher dose of cisplatin in the IC + RT group.

### Baseline characteristics between IC + RT and CCRT groups

The baseline characteristics between IC + RT and CCRT groups showed in Table 1. Before propensity-score matching, there were no significant differences between the two groups regarding the age, gender, T classification, nasopharynx dose, lymph node dose, RT days, basicranial bone involvement and family history. Compared with the CCRT group, the IC + RT group had significantly more patients developed cranial nerve involvement (20.7 % VS. 6.8 %, *P* = 0.037), showed significantly more advanced clinical stage (54.1 % VS. 40.9 %, *P* = 0.048), and N classification (20.7 % VS. 4.5 %, *P* = 0.018). With the use of propensity-score matching (1:2), 44 patients who underwent CCRT were matched with 88 patients who underwent IC + RT. After matching, the balance improvement of the mean differences for all variables were 29.8 %, and baseline characteristics between the two groups were well balanced (Table 1).

**Table 1:** Baseline characteristics before and after propensity-score matching between IC + RT and CCRT groups

Characteristics	Before Matching		*P*	After Matching		*P*
	IC + RT(*N* = 111)	CCRT(*N* = 44)		IC + RT(*N* = 88)	CCRT(*N* = 44)	
Age (y)			0.900			0.506
Mean	64.09	64.59		64.16	64.59	
SD	3.20	4.06		3.20	4.06	
Gender (%)			0.059			1.000
Male	95(85.6)	38(86.4)		76(86.4)	38(86.4)	
Female	16(14.4)	6(13.6)		12(13.6)	6(13.6)	
T-stage (%)			0.817			0.404
T1	4(3.6)	1(2.3)		4(4.5)	1(2.3)	
T2	21(18.9)	11(25.0)		16(18.2)	11(25.0)	
T3	44(39.6)	16(36.4)		36(40.9)	16(36.4)	
T4	42(37.8)	16(36.4)		32(36.4)	16(36.4)	
N-stage (%)			0.018			0.068
N0	19(17.1)	11(25)		18(20.5)	11(25)	
N1	24(21.6)	17(38.6)		20(22.7)	17(38.6)	
N2	45(40.5)	14(31.8)		40(45.5)	14(31.8)	
N3	23(20.7)	2(4.5)		10(11.4)	2(4.5)	
Clinical stage (%)			0.048			0.175
II	4(3.6)	6(13.6)		4(4.5)	6(13.6)	
III	47(42.3)	20(45.5)		43(48.9)	20(45.5)	
IV	60(54.1)	18(40.9)		41(46.6)	18(40.9)	
NP dose (Gy)			0.725			0.704
Mean	71.03	71.23		71.00	71.23	
SD	3.07	3.48		3.11	3.48	
LN dose (Gy)			0.054			0.230
Mean	61.87	59.83		61.21	59.83	
SD	5.79	6.26		6.13	6.26	
RT days			0.390			0.428
Mean	45.16	47.25		48.53	47.25	
SD	8.79	8.32		8.95	8.32	
CNI (%)			0.037			0.245
Present	23(20.7)	3(6.8)		12(13.6)	3(6.8)	
Absent	88(79.3)	41(93.2)		76(86.4)	41(93.2)	
BBI (%)			0.387			0.458
Present	47(42.3)	22(50.0)		38(43.2)	22(50.0)	
Absent	64(57.7)	22(50.0)		50(56.8)	22(50.0)	
Family history (%)			0.632			0.907
Present	6(5.4)	4(9.1)		6(6.8)	4(9.1)	
Absent	105(94.6)	40(90.9)		82(93.2)	40(90.9)	

*IC* + *RT* induction chemotherapy followed by radiotherapy, *CCRT* concurrent chemoradiotherapy, *NP* nasopharynx, *LN* lymph node, *CNI* Cranial nerve involvement, *BBI* Basicranial Bone involvement, *SD* standard deviation

### Survival in the propensity score-matched cohort

**Fig. 1:**
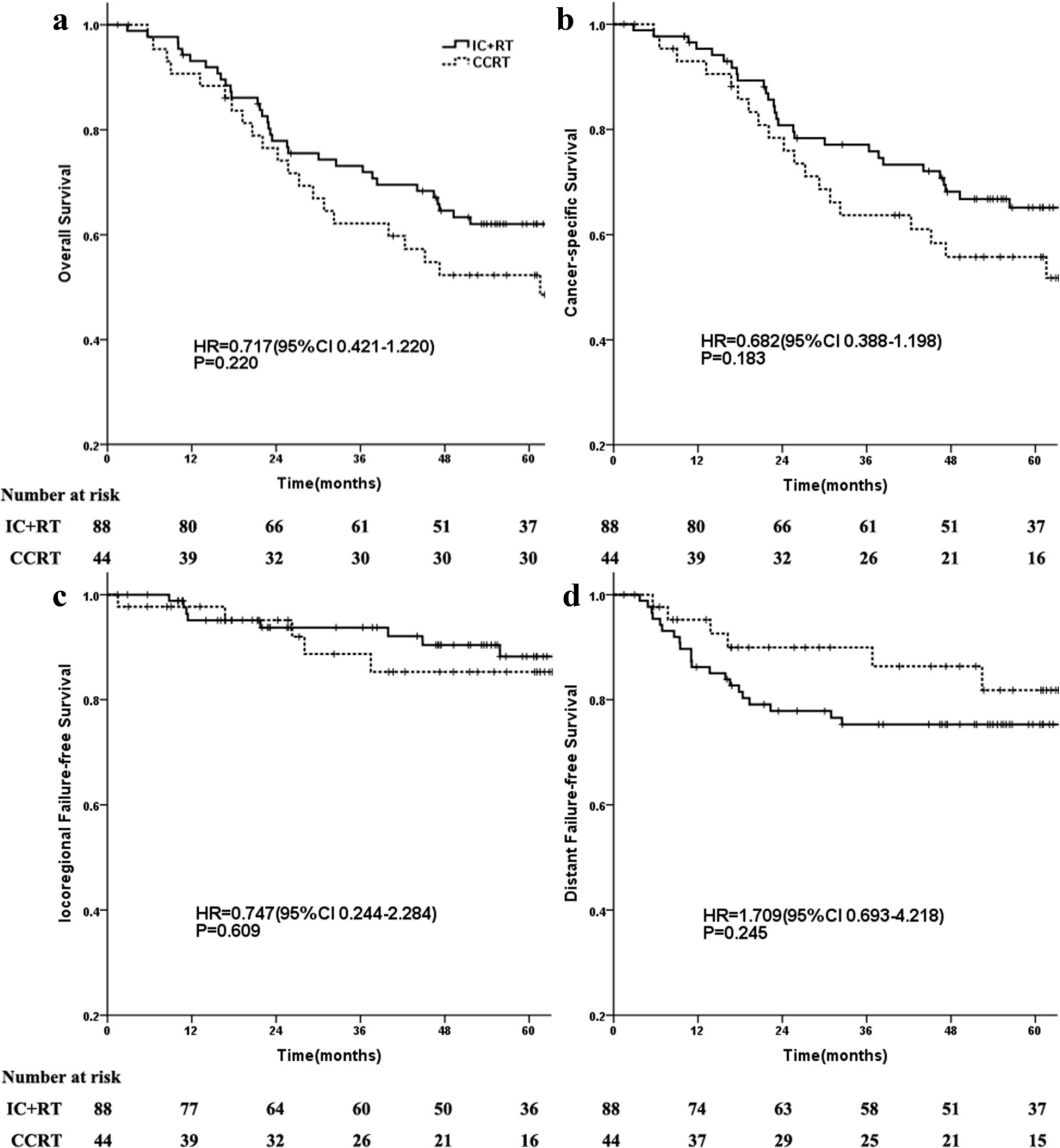
Kaplan-Meier survival curves for the IC + RT and CCRT groups. Notes: Overall survival (**a**), Cancer-specific survival (**b**), Locoregional failure-free survival (**c**), and distant failure-free survival (**d**); Hazard ratios (HRs) were calculated with the unadjusted Cox proportional hazards model; *P* values were calculated with the unadjusted log-rank test. CCRT:concurrent chemoradiotherapy; IC + RT: induction chemotherapy followed by radiotherapy alone. The supplementary dataset file shows the data used in our study, including age group, family history, VCA/EA-IgA, clinical stage, T stage, N stage, RT dose, cranial nerve involvement, basicranial bone involvement, treatment group

As shown in Fig. 1, The 5-year OS for the IC + RT and CCRT groups were 62.1 % and 52.3 % (*P =* 0.218, Fig. 1a), respectively. The 5-year CSS rate in the IC + RT group was 65.2 % compared with 55.7 % in the CCRT group (*P =* 0.180, Fig. 1b). The 5-year LR-FFS for the IC + RT and CCRT groups were 88.2 % and 85.3 % (*P =* 0.607, Fig. 1c), respectively. The 5-year D-FFS rate in the IC + RT group was 75.3 % compared with 81.8 % in the CCRT group (*P =* 0.239, Fig. 1d). These results showed no significant differences were found between the two groups in OS, FFS, LR-FFS, or D-FFS.

To further clarify the role of IC and CCRT in NPC, Patients received sufficient cycles of IC (*n* = 73) and CCRT (*n* = 30) were compared using the propensity score matching. Similarly, baseline characteristics were well matched after propensity score matching (Additional file 1: Table S1). Still, no survival benefits were observed between IC + RT and CCRT groups for 5-year OS (65.6 % VS. 57.0 %, *P* = 0.332), CSS (66.7 % VS. 59.1 %, *P* = 0.332), LR-FFS (88.4 % VS. 84.3 %, *P* = 0.545), and D-FFS (81.6 % VS. 71.9 %, *P* = 0.952).

### Univariate and multivariate analysis in the propensity score-matched cohort

As shown in Table 2, in the univariate analysis, treatment group(IC + RT vs. CCRT) was not associated with survival; basicranial bone involvement was significant factor that predicted OS (HR = 0.553; 95 % CI 0.329–0.929; *P* = 0.025) and CSS (HR = 0.558; 95 % CI 0.321–0.969; *P* = 0.038). After adjustment for age (continuous variable), gender (male vs. female), T classification (T1-2 vs. T3-4), N classification (N0-1 vs. N2-3), clinical stage (I-II vs. III-IV), nasopharynx dose (continuous variable), lymph node dose (continuous variable), cranial nerve involvement(absent vs. present), basicranial bone involvement(absent vs. present) and family history(absent vs. present), treatment group(IC + RT vs. CCRT) still failed to predict OS (HR = 0.706; 95 % CI 0.412–1.208; *P* = 0.204), CSS (HR = 0.708; 95 % CI 0.402–1.246; *P* = 0.231), LR-FFS(HR = 0.696; 95 % CI 0.207–2.342; *P* = 0.558), and D-FFS(HR = 1.627; 95 % CI 0.658–4.023; *P* = 0.292). The significant variable that predicted all-cause death and cancer specific death was basicranial bone involvement. Additionally, T classification was independent prognostic factor that predicted localregional tumor recurrence.

**Table 2:** Univariate and multivariate analyses in patients received IC + RT(*n* = 88) or CCRT(*n* = 44) after propensity score matching

		OS	CSS		LRFFS		DFFS		
		HR (95 % CI)	*P*	HR (95 % CI)	*P*	HR (95 % CI)	*P*	HR (95 % CI)	*P*
Univeriate parameter									
Age	Continuous variable	1.051(0.977–1.131)	0.185	1.046 (0.967–1.132)	0.261	0.892(0.737–1.079)	0.239	1.044(0.945–1.154)	0.399
Gender	male vs. female	1.301(0.590–2.869)	0.514	1.119(0.504–2.487)	0.782	2.131(0.277–16.397)	0.467	1.434(0.433–4.751)	0.555
T-stage	T1-2 vs. T3-4	0.907(0.496–1.659)	0.752	0.974(0.518–1.833)	0.936	2.725(0.915–8.115)	0.072	0.984(0.418–2.320)	0.971
N-stage	N0-1vs. N2-3	0.902(0.538–1.512)	0.902	1.087(0.628–1.883)	0.765	1.200(0.403–3.570)	0.744	0.763(0.361–1.614)	0.479
Clinical stage	I-II vs. III-IV	0.805(0.291–2.228)	0.805	0.929(0.334–2.580)	0.887	2.088(0.462–9.430)	0.338	0.436(0.059–3.209)	0.415
NP dose (Gy)	Continuous variable	1.007(0.930–1.090)	0.862	1.002(0.921–1.090)	0.960	0.925(0.808–1.058)	0.253	1.042(0.923–1.175)	0.510
LN dose (Gy)	Continuous variable	1.023(0.980–1.068)	0.292	1.021(0.975–1.068)	0.380	0.946(0.871–1.027)	0.187	1.057(0.993–1.125)	0.083
RT Days	Continuous variable	1.006(0.981–1.032)	0.623	1.010(0.984–1.037)	0.451	0.954(0.887–1.026)	0.203	1.013(0.977–1.050)	0.493
Treatment group	IC + RT vs. CCRT	0.717(0.421–1.220)	0.220	0.682(0.388–1.198)	0.183	0.747(0.244–2.284)	0.609	1.709(0.693–4.218)	0.245
Cranial nerve involvement	absent vs. present	0.781(0.354–1.726)	0.542	0.663(0.298–1.476)	0.314	0.581(0.128–2.628)	0.481	0.657(0.228–1.896)	0.437
Basicranial Bone involvement	absent vs. present	0.553(0.329–0.929)	0.025	0.558(0.321–0.969)	0.038	0.784(0.263–2.341)	0.663	0.712(0.339–1.497)	0.371
Family history	absent vs. present	1.142(0.413–3.155)	0.798	1.349(0.420–4.335)	0.615	22.74(0.003–25.96)	0.494	0.655(0.197–2.171)	0.489
Multivariate parameter^a^									
Treatment group	IC + RT vs. CCRT	0.706(0.412–1.208)	0.204	0.708(0.402–1.246)	0.231	0.696(0.207–2.342)	0.558	1.627(0.658–4.023)	0.292
Basicranial Bone involvement	absent vs. present	0.553(0.329–0.929)	0.025	0.558(0.321–0.969)	0.038	0.246(0.044–1.382)	0.111	0.760(0.337–1.715)	0.508
T-stage	T1-2 vs. T3-4	1.347(0.638–2.842)	0.435	1.490(0.675–3.289)	0.324	6.833(1.224–38.148)	0.028	1.355(0.481–3.819)	0.565

*CI* confidence interval, *IC* + *RT* induction chemotherapy followed by radiotherapy, *CCRT* concurrent chemoradiotherapy, *NP* nasopharynx, *LN* lymph node

^a^Other covariates not shown (*P* > 0.05)

### Treatment toxicities

To compare the incidence of treatment toxicities between IC + RT and CCRT groups, patients received sufficient courses of IC + RT or CCRT were chose. As listed in Table 3. Regarding hematologic toxicities, incidences of grade III and IV leukopenia (30 % vs. 6.8 %, *P* = 0.005), anemia (20 % vs. 4.1 %, *P* = 0.027) and granulocytopenia (26.7 % vs. 5.5 %, *P* = 0.007) were significantly higher in the CCRT group. No significant difference in thrombocytopenia (13.3 % vs. 2.7 %, *P* = 0.105) was found between the two groups. With respect to nonhematologic toxicity, the incidences of grade III and IV mucositis (63.3 % vs. 34.2 %, *P* = 0.007), skin reaction (20.0 % vs. 4.1 %, *P* = 0.027), and weight loss (23.4 % vs. 4.1 %, *P* = 0.027) were significantly higher in the CCRT group; while no significant differences were detected regarding the incidence of severe vomiting and hepatic impairment between the groups. In addition, no severe renal toxicity was seen in either group. Late toxicities were also analyzed in our study. Unlike acute toxicities, the incidence of severe late toxicities was comparable between both groups (Table 3).

**Table 3:** Incidences of serious toxicities during radiotherapy course between IC + RT and CCRT groups

Toxicity	IC + RT (%,*N* = 73)		CCRT (%,*N* = 30)		*P*
	Grade 3	Grade 4	Grade 3	Grade 4	
Acute toxicity					
Leukopenia	5(6.8)	0	7(23.3)	2(6.7)	0.005
Granulocytopenia	4(5.5)	0	6(20.0)	2(6.7)	0.007
Thrombocytopenia	2(2.7)	0	3(10.0)	1(3.3)	0.105
Anemia	2(2.7)	1(1.4)	4(13.3)	2(6.7)	0.027
Vomiting	0	0	2(6.7)	0	0.149
Mucositis	25(34.2)	0	16(53.3)	3(10.0)	0.007
Skin reaction	3(4.1)	0	5(16.7)	1(3.3)	0.027
Hepatic impairment	1(1.4)	0	1(3.3)	0	1.000
Renal impairment	0	0	0	0	
Weight loss	3(4.1)	0	5(16.7)	2(6.7)	2(6.7)
Late toxicity					
Xerostomia	3(4.1)	0	2(6.7)	0	0.965
Subcutaneous Fibrosis	5(6.8)	0	2(6.7)	0	1.000
Temporal lobe necrosis	2(2.7)	0	1(3.3)	0	1.000
Trismus	2(2.7)	0	0	0	0.897
Dysphagia	3(4.1)	0	2(6.7)	0	0.965
Cranial neuropathy	1(1.4)	0	0	0	1.000

*IC* + *RT* induction chemotherapy followed by radiotherapy, *CCRT* concurrent chemoradiotherapy

## Discussion

Numerous studies were carried out to address the use of chemotherapy in combination with RT for the care of locoregionally advanced NPC (which involved only a few elderly patients). A 2012 meta-analysis [12] which included six trials in IC + RT group (*n* = 1418) and five in AC group (*n* = 1187) found that IC + RT can effectively enhance OS and reduce the risk of distant failure. However, a recent another meta-analysis [13] that included 19 trials and 4806 patients confirmed the addition of chemotherapy to radiotherapy significantly improved OS in favor of CCRT + AC and CCRT without AC but not AC alone or IC + RT alone. To date, it is generally believed that CCRT is the most efficacious modality for non-elderly patients. In contrast, previous studies for elderly NPC patients have shown either IC + RT or CCRT can improve the survival of elderly NPC patients [3, 5]. But which is a favorable regimen remains unclear, it is necessary to elucidate the roles of IC + RT or CCRT in elderly NPC patients given the poor compliance with combined chemoradiotherapy, especially CCRT.

In the present propensity-matched study, the results confirmed our hypothesis. No significant differences between IC + RT and CCRT groups were found regarding overall survival, cancer-specific survival, locoregional failure-free survival, or distant failure-free survival. Patients received sufficient cycles of IC (*n* = 73) and CCRT (*n* = 30) were further compared using the propensity-matched analysis. We found that 5-year OS, CSS,and D-FFS were higher in the IC + RT group compared with CCRT group, but the difference was not statistical significance(OS:65.6 % VS. 57.0 %, *P* = 0.332; CSS: 65.6 % VS. 57.0 %, *P* = 0.332; D-FFS: 81.6 % VS. 71.9 %, *P* = 0.952). This was mainly due to the relatively small matched pairs even using 1:2 matching on the propensity score (Additional file 1: Table S1).

The most probable explanation for this negative result might either IC + RT or CCRT can improve the locoreginal control, but failed to further decrease the distant metastasis compared with radiotherapy alone. NPC is a highly chemosensitive solid tumor [14]. Induction chemotherapy can increase tumor sensitivity to radiation through shrinking the primary tumor and improving the intratumoral blood supply and re-oxygenation, which also lead to an increased safety margin between the radiation volume and the tumor volume [15, 16]; For patients received CCRT, the synergistic effects between cytotoxic agents and radiation can also improve the locoreginal control of the primary tumor [17]. Thus, the radiosensitizing effect of chemotherapy is similar in patients received IC + RT or CCRT. However, neither IC + RT nor CCRT can further improve the D-FFS in elderly patients, which is mainly because the elderly patients have worse compliance with combined chemoradiotherapy compared to the non-elderly patients [3, 5]. In addition, the effective of chemotherapy is involved with dose intensity, but our data showed that elderly NPC patients often received fixed lower total doses of each drug irrespective of body surface area, mainly as a result of arbitrary dose modification of chemotherapy owing to fear of excessive side-effects, which was also seen in the other studies [18, 19]. In clinical practice, because there were no proposed guidelines for elderly NPC patients, oncologists often attached importance to the treatment-related toxicities and preferred a lower dose without evaluation. As a consequence, this conservative treatment selection potentially prevented some elderly patients from longer survival [5]. More importantly, distant metastases remain the predominant pattern of treatment failure in NPC patients [20], previous studies have shown even IC + CCRT failed to decrease the distant metastases [6, 7]. Geriatric oncologists should exploit other advances made in the management of non-elderly NPC, such as the addition of targeted agents to chemoradiotherapy [21, 22], which have obtained some promising outcomes (2-year D-FFS of about 90 %).

It is generally accepted that the elderly cancer patients experienced an increased treatment-induced toxicity [19, 23]. Some reasons accounting for this included more common comorbidities [24], an increased exposure to a drug (e.g. by impaired renal function or by prolonged half-life due to decreased elimination) and changes in pharmacodynamics caused by increased vulnerability of organs with age [25]. However, previous studies shown the rates of severe acute and late toxicities caused by CCRT in elderly patients were similar with younger patients [5, 26]. It is likely that a selection and referral bias in these studies lead to accrual of only fit elderly patients [25]. In the present study, the toxicities in elderly patients received sufficient courses of IC + RT or CCRT were compared. Although the incidence of severe late toxicities was comparable between both groups, patients received CCRT were associated with more acute toxicities, as compared with patients received IC + RT, including leucopenia, granulocytopenia, anemia, mucositis, skin reaction, weight loss (Table 3). The high incidence of severe acute toxicities in CCRT group may interrupt oncologic treatment, increase the risk of unplanned hospitalization, and seriously affect the quality of life in elderly patients [27, 28]. Thus, geriatric oncologists should pay more attention to elderly NPC patients received CCRT in future.

In spite of no significant survival differences between CCRT and IC + RT groups, the entire patient cohort was analyzed to identify valuable prognostic factors in the elderly NPC patients. Multivariate analysis showed basicranial bone involvement remained an independent prognostic factor that predicted all-cause death and cancer specific death in elderly patients and T classification predicted localregional tumor recurrence. Contrary to several non-elderly series [29–31], age, gender, N classification, and family history failed to predict all survival endpoints for elderly patients. The results suggested the potentially different clinical characteristics between the elderly patients and their younger ones.

To the best of our knowledge, there is very little published information regarding the optimal chemotherapy modalities of elderly NPC. In the past, the elderly NPC patients were treated very differently at different cancer centers. Our intention was not to test a novel therapy but to ensure an equivalent therapeutic effect and less treatment-induced toxicities for the elderly patients. Some limitations in our study should be considered. Firstly, this was a nonrandomized, retrospective study and hence suffered from potential selection bias despite robust propensity-score matching. Secondly, comorbidities were not further assessed, which may have effect on survival, although cancer-specific survival was used to exclude death due to comorbidities. Finally, all patients were treated using conventional RT technique, whether it is preferable to combine chemotherapy and intensity-Modulated Radiation Therapy (IMRT) should be investigated in future.

## Conclusions

In summary, the present propensity-matched study demonstrated the elderly NPC patients received IC + RT achieve similar survival outcomes compared with patients received CCRT, but with less treatment-induced acute toxicities. In the context of no guideline for elderly NPC, the present study suggested IC + RT should be a preferable modality compared with CCRT. It is hoped that the current outcomes could provide a more accurate basis for designing future clinical trials.

## Abbreviations

CCRT, concurrent chemoradiotherapy; CRT, combined chemo-radiotherapy; CSS, cancer-specific survival; CTCAE, the Common Terminology Criteria for Adverse Events; D-FFS, distant failure-free survival; ECT, single photon emission computed tomography; HR, hazard ratio; IC + RT, induction chemotherapy followed by radiotherapy; LRFFS, local-regional failure-free survival; NPC, Nasopharyngeal carcinoma; OS, overall survival; RT, radiotherapy

## Additional files

Additional file 1: Table S1. Baseline characteristics before and after propensity-score matching in patients received sufficient cycles of IC + RT and CCRT. (DOC 95 kb) Additional file 2: List of elderly patients with nasopharyngeal carcinoma in our study. (XLSX 49 kb)
