# Supplementary material for: Induction Chemotherapy Followed by Radiotherapy versus Concurrent Chemoradiotherapy in elderly patients with nasopharyngeal carcinoma: finding from a propensity-matched analysis
Source: BMC Cancer. 2016 Aug 30;16(1):693. doi: 10.1186/s12885-016-2661-y (PMC5004294; doi:10.1186/s12885-016-2661-y)
Supplement: Additional file 1: Table S1. — Baseline characteristics before and after propensity-score matching in patients received sufficient cycles of IC + RT and CCRT. (DOC 95 kb) [file 12885_2016_2661_MOESM1_ESM.doc]

**Supplementary table 1. Baseline characteristics before and after propensity-score matching in patients received sufficient cycles of IC+RT and CCRT.**

|  | **Before Matching** | |  | **After Matching** | |  |
| --- | --- | --- | --- | --- | --- | --- |
| **Characteristics** | IC+RT(N=73) | CCRT(N=30) | P | IC+RT(N=60) | CCRT(N=30) | P |
| **Age(y)** |  |  | 0.471 |  |  | 0.559 |
| mean | 64.22 | 63.70 |  | 64.13 | 63.70 |  |
| SD | 3.19 | 3.34 |  | 3.20 | 3.34 |  |
| **Gender (%)** |  |  | 0.401 |  |  | 1.000 |
| male | 62(84.9) | 28(93.3) |  | 55(91.7) | 28(93.3) |  |
| Female | 11(15.1) | 2(6.7) |  | 5(8.3) | 2(6.7) |  |
| **T-stage (%)** |  |  | 0.766 |  |  | 0.929 |
| T1 | 3(4.1) | 1(3.3) |  | 3(5.0) | 1(3.3) |  |
| T2 | 12(16.4) | 7(23.3) |  | 11(18.3) | 7(23.3) |  |
| T3 | 27(37.0) | 11(36.7) |  | 24(40.0) | 11(36.7) |  |
| T4 | 31(42.5) | 11(36.7) |  | 22(36.7) | 11(36.7) |  |
| **N-stage (%)** |  |  | 0.088 |  |  | 0.101 |
| N0 | 15(20.5) | 9(30.0) |  | 13(21.7) | 9(30.0) |  |
| N1 | 14(19.2) | 10(33.3) |  | 11(18.3) | 10(33.3) |  |
| N2 | 27(37.0) | 9(30.0) |  | 22(36.7) | 9(30.0) |  |
| N3 | 17(23.3) | 2(6.7) |  | 14(23.3) | 2(6.7) |  |
| **Clinical stage(%)** |  |  | 0.149 |  |  | 0.297 |
| Ⅱ | 2(2.7) | 3(10.0) |  | 2(3.3) | 3(10.0) |  |
| Ⅲ | 28(38.4) | 14(48.7) |  | 25(41.7) | 14(48.7) |  |
| Ⅳ | 43(58.9) | 13(43.3) |  | 33(55.0) | 13(43.3) |  |
| **NP dose(Gy)** |  |  | 0.145 |  |  | 0.154 |
| mean | 70.79 | 71.87 |  | 70.73 | 71.87 |  |
| SD | 3.10 | 3.93 |  | 3.31 | 3.93 |  |
| **LN dose(Gy)** |  |  | 0.031 |  |  | 0.062 |
| mean | 61.56 | 58.54 |  | 61.33 | 58.54 |  |
| SD | 6.16 | 6.88 |  | 6.44 | 6.88 |  |
| **RT days** |  |  | 0.084 |  |  | 0.086 |
| mean | 48.75 | 45.77 |  | 48.85 | 45.77 |  |
| SD | 7.58 | 7.91 |  | 7.94 | 7.91 |  |
| **CNI (%)** |  |  | 0.200 |  |  | 1.000 |
| present | 15(20.5) | 3(10.0) |  | 7(11.7) | 3(10.0) |  |
| absent | 58(79.5) | 27(90.0) |  | 53(88.3) | 27(90.0) |  |
| **BBI(%)** |  |  | 0.485 |  |  | 0.549 |
| present | 31(42.5) | 15(50.0) |  | 26(43.3) | 15(50.0) |  |
| absent | 42(57.5) | 15(50.0) |  | 34(88.3) | 15(50.0) |  |
| **Family history(%)** |  |  | 0.486 |  |  | 0.654 |
| present | 3(4.1) | 3(10.0) |  | 3(5.0) | 3(10.0) |  |
| absent | 70(95.9) | 27(90.0) |  | 57(95.0) | 27(90.0) |  |

Notes: IC+RT: induction chemotherapy followed by radiotherapy; CCRT: concurrent chemoradiotherapy; NP:

nasopharynx; LN: lymph node; CNI: Cranial nerve involvement; BBI: Basicranial Bone involvement
